# Management of Femoral Shaft Fractures: The Significance of Traction or Operative Position

**DOI:** 10.7759/cureus.33776

**Published:** 2023-01-14

**Authors:** Dhruv Gupte, Daniel Axelrod, Tanis Worthy, Taylor Woolnough, Asher Selznick, Herman Johal

**Affiliations:** 1 Orthopedic Surgery, Schulich School of Medicine & Dentistry, University of Western Ontario, London, CAN; 2 Orthopedic Surgery, McMaster University, Hamilton, CAN

**Keywords:** surgical positioning, free leg, traction table, femoral shaft fracture, antegrade intramedullary nail, orthopaedic trauma surgery, orthopaedic surgery

## Abstract

Background and objective

Intramedullary femoral nailing (IMN) is the gold standard for managing femoral shaft fractures (FSFs). Though good clinical outcomes and union rates have been reported following this procedure, it has also been commonly associated with perioperative complications. Positioning the patient in lateral decubitus, avoiding a fracture table, or using manual traction have been touted as possible techniques to reduce perioperative complications in IMN. However, given the scarce availability of comparative research, the decision to employ any of the techniques mentioned above is often guided by surgeon preference alone. In light of this, the purpose of this study was to determine whether the use of free-leg draping using either supine or direct lateral positioning with manual traction reduces perioperative complications among trauma patients undergoing an anterograde femoral nailing surgery when compared to using a fracture table.

Methods

Consecutive adult patients from a level-one trauma center undergoing unilateral antegrade femoral fixation surgeries between 2016 and 2020 were retrospectively evaluated for possible inclusion in the study. Relevant perioperative and postoperative data, including length of hospital or ICU stay and perioperative complications, were included in the analysis. This study received research ethics board approval before data collection began.

Results

A total of 91 patients were ultimately included in the final analysis: 61 patients were included in the free-leg draping with manual traction group (FL) and 30 patients were included in the traction table group (TT). Patients in the FL group had a similar operative and fluoroscopy time, blood loss, length of stay, and time on the ventilator. Subgroup analysis comparing positioning within the FL group revealed non-significant differences in fluoroscopy time (p=0.59) and length of stay (p=0.20) between the lateral and supine groups. Moreover, no differences in operative time, blood loss, and time on the ventilator were observed between lateral and supine groups.

Conclusion

Based on our findings, there were no significant differences in terms of operative or fluoroscopy time, perioperative complications, or length of time in the hospital or on the ventilator between the groups. Our study was limited by its small sample size and incomplete data. Further prospective randomized research is required to reach definitive conclusions on the appropriate manner to treat patients with these complex and morbid injuries.

## Introduction

Femoral shaft fractures (FSFs) are severe injuries that can cause substantial mortality and morbidity in the affected patients if not managed appropriately [1-3]. Femur fractures can lead to extensive bleeding and muscle injury of the thigh and are associated with a significant healthcare burden worldwide, occurring at a rate between 14 and 42.5/100,000 person-years, and accounting for 19-35% of all motor vehicle accidents [4-6].

Currently, intramedullary femoral nailing (IMN) is the gold standard for the surgical management of FSF [1,6-8]. Although the union rate following intramedullary fixation of FSFs is 97% [9], the procedure is also commonly associated with adverse events such as postoperative pain, femoral malrotation, prolonged hospital stay, and gait dysfunction [10-12].

Altered patient positioning (either lateral or supine) and the use or avoidance of a fracture table have been touted separately as possible techniques to reduce perioperative complications [13,14]. However, a recent multinational survey on this topic revealed significant heterogeneity in terms of the practice of these techniques among orthopedic surgeons [15]. While the vast majority of community orthopedic surgeons (82%) preferred the use of a traction table (which most often places the patient in a supine position), a slight majority of academic or level one trauma surgeons preferred lateral or sloppy lateral positioning with manual traction (60%). Moreover, the distribution varied between surgeons located in Canada and the US, with 89% and 27% preferring a fracture table and supine positioning in Canada and the US respectively. For patients with C-spine injuries or those with an airway compromise, supine positioning may also be the more favored option with regard to the delivery of anesthetic care [16,17].

Supine positioning during an antegrade IMN is often accompanied by a fracture (or traction) table [14,18]. Injured limbs are secured in a set position using a fracture table through a combination of boot or skeletal traction pins, providing variable degrees of mechanical traction, and posts and straps maintaining countertraction [19]. Despite being able to achieve excellent length, malreduction may still occur if the use of a traction table overcomes the patient’s resting soft tissue tension [20]. Subsequent malalignment and malrotation can further complicate patient outcomes. Prolonged use of traction has also been associated with nerve palsies and iatrogenic compartment syndrome impacting the non-affected limb, as well as a perineal soft-tissue compromise [20]. These are all serious and potentially avoidable complications that must be thoughtfully considered before using a traction table.

Whereas supine positioning with a fracture table was long seen as the standard of care, there has been a slow uptake in surgeons willing to perform femoral IMN in lateral or sloppy lateral positioning [21]. This reluctance may stem from the belief that lateral positioning can compromise airway function and prolong both extubation and ICU stay, especially in patients with multiple injuries [22]. However, the literature does not support this perception and instead indicates that when patients are placed laterally, ICU stay and days on a ventilator may actually be reduced [23,24]. Additionally, improved access to point of entry with obese patients, a potential reduction in traction table-related soft-tissue injuries, and increased cost and time savings by eliminating the need for a traction table are some of the proposed benefits of lateral positioning with manual traction [17,19,20,25-30].

Surgeons may also be more experienced in, and therefore more comfortable with, operating on FSFs with a fracture table and supine positioning. This surgical technique is adaptable for other fixation surgeries as well, such as femoral neck fractures and intertrochanteric hip fractures, thereby enhancing the surgeon's familiarity with this approach. Hesitancy towards placing patients laterally may also be attributed to the fact that intraoperative fluoroscopic views differ greatly between supine and lateral positions. For surgeons with less experience in operating using the lateral position during IMN, interpreting these images may be challenging and can lead to the perception that lateral positioning during these procedures prolongs iatrogenic radiation exposure [26]. However, good radiographic visualization of the femur is still attainable in the lateral position [26].

Despite the availability of current research with regard to appropriate surgical techniques for IMN procedures, there is a lack of consensus as to which technique is the most effective at reducing adverse events and outcomes. Hence, the purpose of this study was to determine whether the use of free-leg draping in either supine or direct lateral position with manual traction reduces perioperative complications compared to intraoperative traction using a traction table in orthopedic trauma patients undergoing antegrade femoral nailing surgery.

## Materials and methods

Study population

This retrospective study involved patients undergoing unilateral antegrade femoral fixation surgeries at a level-one trauma center [Hamilton General Hospital (HGH)] in Ontario, Canada between January 2016 and December 2020. The choice of January 2016 as the starting point was due to the fact that prior to this no surgeons at HGH were performing IMN surgeries in the lateral position. The Inclusion criteria were as follows: patients aged 16 years or older who underwent antegrade fixation using an IMN for a unilateral injury after sustaining an acute non-pathologic fracture. The exclusion criteria were as follows: patients who underwent retrograde nailing, whose surgeries involved short IM nail (without distal locking screw), and those with femoral head fractures, femoral neck fractures treated with Dynamic Hip Screw (DHS), femoral exchange nailing, and those who received prophylactic fixation for impending fractures.

Patients included were subsequently number-coded and were only identifiable via their patient numbers to ensure confidentiality. Patient-important outcomes were abstracted for by a team of six independent reviewers. Spot checks were undertaken after the completion of the review of cases in each of the four years of the study period by two senior reviewers (DA, HJ). All data were reassessed, cleaned, and reformatted by one reviewer once abstraction was completed.

Patients undergoing surgery using a traction table (TT group) were compared to those with the free-leg draping technique and manual traction (FL group). Further analysis within the free-leg draping population was also conducted with regard to patient positioning (supine or direct lateral). Data related to the following variables were analyzed: the length of procedure, need for adjuncts, blood loss, intraoperative radiation exposure (measured through fluoroscopy time), need and reason for reoperation, postoperative respiratory status, days on the ventilator, length of ICU admission (if applicable), length of hospital stay, time to bony union, length of follow up, postoperative mobilization, and mortality.

**Figure 1:**
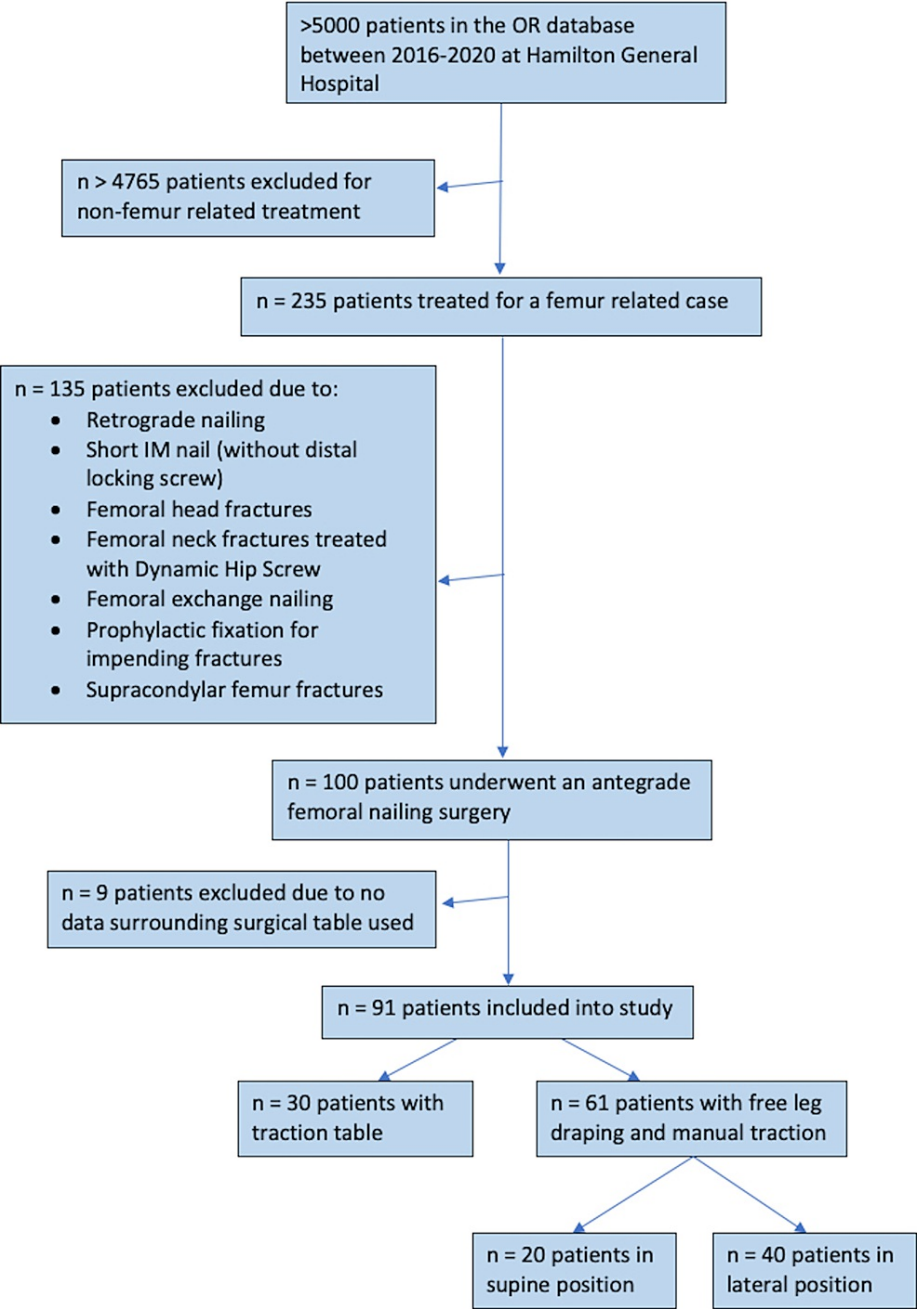
Chart depicting the patient selection process Selection of patients for the study from the HGH OR database and subsequent classification into different groups (traction table with supine and free-leg draping with manual traction in either supine or lateral positions)

This study received institutional research ethics board approval from the Hamilton Research Ethics Board (HiREB# 4935). Figure 1 depicts the process of patient inclusion in the study.

Statistical analysis

Simple descriptive statistics were collected and analyzed through Microsoft Excel (Microsoft Corporation, Redmond, WA). Categorical variables were analyzed using a two-tailed Fisher’s exact test. For continuous variables, data were presented as means and standard deviations (SD). Given the explorative and retrospective nature of this study, no sample size was determined a priori.

## Results

A total of 235 patients who underwent a femoral nailing surgery between 2016 and 2020 were screened through chart review. Among them, 100 patients met the initial inclusion criteria; however, nine patients were excluded due to a lack of data regarding the table used. Hence, 91 patients were ultimately included in the sample. The mean age of the sample was 50.7 ± 23.9 years; 57 patients were male and 34 were female. Within the sample, 35 patients had polytraumas, with 33 patients undergoing combined surgeries with additional orthopedic procedures. Postoperatively, nine patients required intubation, and 16 were transferred to the ICU. The average follow-up time for the sample was 270 days. The sample was retrospectively divided into the FL group (n=61, mean age: 55 years, 43.33% women) and the TT group (n=30, mean age: 49 years, 34.43% women) (Table 1). Patients in the FL group were then subdivided based on their positioning - supine (n=20) vs. lateral (n=40) - with one patient having no available data on positioning (Table 2).

**Table 1:** Fracture table vs. free leg with manual traction Comparison of descriptive statistics between patients undergoing an antegrade femoral IMN surgery using a traction table and those who underwent manual traction with free-leg draping SD: standard deviation; IMN: intramedullary nailing; ICU: intensive care unit

Variables	Fracture table (n=30)	Free leg with manual traction (n=61)
Lateral position	5	40
Supine position	22	20
Age, years, mean ± SD	54.866 ± 24.53	48.64 ± 23.54
Female, %	43.33%	34.43%
Left side, %	46.67%	50.82%
Length of stay, days, mean ± SD	17.4 ± 17.14	14.81 ± 16.21
Associated lung injury, %	20%	31.15%
Polytrauma, %	46.67%	49.18%
Isolated femoral nailing, %	70%	60.66%
Length of procedure, minutes, mean ± SD	130.2 ± 65.88	139.19 ± 80.62
Need for adjuncts, %	13.33%	27.87%
Blood loss, mL, mean ± SD	351.388 ± 209.07	301.97 ± 216.88
Fluoroscopy time, seconds, mean ± SD	262.57 ± 183.57	319.78 ± 150.38
Postop location ICU, %	26.67%	14.75%
Postop resp status intubated, %	10%	14.75%
Need for reoperation, %	20%	13.11%
Ventilator use, days, mean ± SD	1.758 ± 5.309	1.116 ± 3.059
Time to healing, days, mean ± SD	119.9 ± 79.28	126.95 ± 105.21
Length of follow-up, days, mean ± SD	204.95 ± 192.84	297.30 ± 319.74
Mortality, %	23.33%	1.64%

**Table 2:** Free-leg draping with manual traction: lateral vs. supine Comparison of descriptive statistics between patients positioned laterally and those positioned supine (for undergoing an antegrade IMN surgery with free-leg draping with manual traction) SD: standard deviation; IMN: intramedullary nailing; ICU: intensive care unit

Variables	Lateral (n=40)	Supine (n=20)
Age, years, mean ± SD	50.625 ± 24.761	45.4 ± 21.465
Female, %	37.50%	30%
Left side, %	52.50%	45%
Length of stay, days, mean ± SD	13.538 ± 11.614	17.9 ± 22.932
Associated lung injury, %	32.50%	30%
Polytrauma, %	40%	70%
Isolated femoral nailing, %	67.50%	45%
Length of procedure, minutes, mean ± SD	141.275 ± 86.359	132.95 ± 71.228
Need for adjuncts, %	42.50%	30%
Blood loss, mL, mean ± SD	326 ± 235.9025	256.25 ± 181.573
Fluoroscopy time, seconds, mean ± SD	330.830 ± 144.065	306.175 ± 164.147
Postop location ICU, %	17.50%	15%
Postop intubation, %	15%	15%
Need for reoperation, %	10%	20%
Ventilator use, days, mean ± SD	1.51 ± 3.705	0.4 ± 0.8207
Time to healing, days, mean ± SD	122.852 ± 98.71	134.857 ± 120.302
Length of follow-up, days, mean ± SD	276.628 ± 314.809	337.85 ± 340.127
Mortality, %	2.50%	0%

Traction table vs. free-leg draping with manual traction 

Within both the TT and FL groups, fractures were evenly distributed between the left and right femurs. Of the surgeries performed, 21 patients (70%) in the TT group underwent isolated femoral surgeries vs. 37 patients (60.67%) in the FL group. Both groups had similar proportions of patients presenting with polytraumas to the OR, with six patients (20.00%) in the TT group presenting with associated lung injuries as compared to 19 patients (31.15%) in the FL group. Postoperatively, intubation had to be provided in a similar proportion of patients in both groups, while eight TT patients (26.67%) required a postoperative stay in the ICU compared to nine FL patients (14.75%). Mortality occurred in seven patients (23.33%) in the TT group and one patient (1.64%) in the FL group. Reoperation was required for six (20.00%) and eight patients (13.11%) in the TT and FL groups respectively. Time to healing was similar between groups with the TT group having an average of 120 days to bony union compared to 127 days for the FL group. Lastly, a minimal difference was seen in the duration of follow-up between both groups, with the TT group having patients followed up for an average of 204.95 ± 192.84 days, whereas the FL group had patients follow up for an average of 297.30 ± 319.74 days (Table 1).

Intraoperative comparisons and outcome measures between study groups 

Patients in the TT and FL groups had similar values in terms of operative time (130.2 vs. 139.20 minutes, p=0.57), intraoperative fluoroscopy time (262.57 vs. 319.80 seconds, p=0.15), and estimated blood loss during operations (351.38 vs. 301.97 mL, p=0.42). The difference in length of stay between the TT and FL groups was non-significant (17.4 vs. 14.82 days, p=0.50), and so was the time on the ventilator (1.76 vs. 1.12 days, p=0.55) (Table 3).

**Table 3:** T-test analysis of patient-important outcomes between groups T-test analysis of operative and postoperative patient-important outcomes between fracture table group vs. free-leg draping technique with manual traction group. P-values provided

Variables	Traction Table	Free Leg	P-value
Fluoroscopy time, seconds	262.57	319.8	0.15
Operative time, minutes	130.2	139.2	0.57
Blood loss, mL	351.38	301.97	0.42
Length of stay, days	17.4	14.82	0.50
Time on ventilator, days	1.76	1.12	0.55

Subgroup analysis within the FL group revealed no difference between those positioned laterally and those positioned supine in terms of operative time (132.56 vs. 132.9 minutes, p=0.98), fluoroscopy time (309.75 vs. 291.27 seconds, p=0.59), blood loss (314.39 vs. 323.07 mL, p=0.87), and the time on the ventilator (1.19 vs. 1.0 days, p=0.79). Lastly, the length of stay was non-significantly lower in the lateral group (13.82 days vs. 18.39, p=0.20) (Table 4).

**Table 4:** T-test analysis of patient positioning in the manual traction group T-test analysis of operative and postoperative patient-important outcomes between patient positioning in either lateral or supine in the free-leg draping with manual traction group. P-values provided

Variables	Lateral	Supine	P-value
Fluoroscopy time, seconds	309.75	291.27	0.59
Operative time, minutes	132.56	132.9	0.98
Blood loss, mL	314.39	323.07	0.875
Length of stay, days	13.82	18.39	0.20
Time on ventilator, days	1.19	1.0	0.79

## Discussion

The purpose of this study was to compile and assess a retrospective cohort of consecutive patients treated with either a traction table or free-leg draping with manual traction in antegrade intramedullary fixation for FSFs. Further subgroup analysis was conducted within the FL group between patients positioned laterally and those positioned supine. The goal of this cohort analysis was to assess if intraoperative traction and surgical position yielded substantial differences in patient-important outcomes such as operative time, iatrogenic radiation exposure, blood loss, length of stay, and time on the ventilator.

The primary analysis revealed longer operative and intraoperative fluoroscopy time in the FL group compared to the TT group during IMN, but the findings were not statistically significant; nor were they adjusted for plausible confounding variables such as fracture complexity or surgeon experience. Hence, we cannot say with certainty that the use or avoidance of a traction table during IMN leads to differences in operative and fluoroscopic time, perioperative complications, length of stay, or time on the ventilator when compared to manual traction.

Similarly, lateral positioning showed marginal and insignificant increases in fluoroscopy time when compared to the traction table. This finding is understandable as surgeons may find it difficult to obtain appropriate fluoroscopic images, as the images are rotated 90° to the horizontal plane. Patient positioning in this study showed no significant difference in terms of operative and fluoroscopic time as well as perioperative complications, length of stay, and time on the ventilator. 

As a retrospective review, this study has several limitations. As mentioned above, the choice to pursue lateral or supine positioning with or without the use of a fracture table was likely a non-random choice. Patients in each group may have been biased regarding the severity of their fracture type, surgeon comfort, surgeon skill with the procedure, and the availability of assistants. This significantly limits the generalizability of our findings. Furthermore, all of our outcomes were not found to be statistically different between groups. This may have been due to the fact that our study was underpowered. When assessing our outcomes post-hoc, with a desired power of 0.8, a 95% confidence interval, and an estimated mean difference of one or two days for length of stay (as one example outcome), we would have required over 300 patients to reach the appropriate sample size. This reasoning could be applied to all of our findings.

The study points to parameters that need to be further investigated before definitive findings can be made with regard to a comparison between free-leg draping with manual traction and the use of a traction table in antegrade femoral nailing surgery. Moreover, a cost-effectiveness analysis was not performed in this study, and such an analysis could provide an additional consideration when deciding which technique to use, in terms of the cost associated with the fracture table itself or the unique drapes required when using it.

## Conclusions

Based on our findings, there were no significant differences in operative time, intraoperative fluoroscopy time, and length of stay in the hospital or on a ventilator between the FL and TT groups or between patient positioning within the FL group. Given the complex nature of FSFs with high rates of associated orthopedic injuries, it remains a valuable goal to identify techniques to reduce operative complications and postoperative length of stay. We strongly recommend that randomized controlled trials be conducted, which will minimize bias and allow for a balance of confounding variables between operative groups, in order to achieve definitive results regarding this clinical question.
